# Pertussis diagnostic practices of general practitioners in the Netherlands: A survey study

**DOI:** 10.1080/13814788.2019.1639669

**Published:** 2019-08-13

**Authors:** Jeanne Heil, Jochen W. L. Cals, Henriëtte L. G. ter Waarbeek, Christian J. P. A. Hoebe, Nicole H. T. M. Dukers-Muijrers

**Affiliations:** a Department of Sexual Health, Infectious Diseases and Environmental Health, South Limburg Public Health Service, Heerlen, the Netherlands;; b Department of Medical Microbiology, Care and Public Health Research Institute (CAPHRI), Maastricht University Medical Center (MUMC+), Maastricht, the Netherlands;; c Department of Family Medicine, Care and Public Health Research Institute (CAPHRI), Maastricht University (UM), Maastricht, the Netherlands

**Keywords:** Whooping cough, general practitioners, primary healthcare, general practice

## Abstract

**Background:** Pertussis testing is most important when transmission to vulnerable groups is likely. Patients with signs and symptoms suggestive of pertussis are prevalent in primary care, yet general practitioners’ (GPs) reasons (not) to test for pertussis are largely unknown.

**Objectives:** To evaluate GP-reported diagnostic practices for pertussis, reasons for (not) testing and intentions towards testing among GPs.

**Methods:** A total of 594 Dutch GPs were invited by email to participate in a survey study including a questionnaire reflecting on their pertussis diagnostic practices, reasons for (not) testing and the intention to test for pertussis in the year of 2013. Intention to test was measured as the likelihood to test for eight clinical vignettes.

**Results:** In total, 122 GPs (21%) completed the online questionnaire. Most GPs reported having diagnosed at least one pertussis case (84%) in the previous year. Of all GPs, 14% did not perform any pertussis tests in the last year. The most reported reason for testing was to confirm the clinical pertussis diagnosis (70%); the most reported reason for not testing was that the diagnostic test result does not influence treatment (70%). Overall, judging from the clinical vignettes, GPs reported being more likely to perform diagnostic testing based on symptoms than based on vulnerable groups at risk.

**Conclusion:** In contrast to national guidelines, our results suggest that GPs report to test for pertussis mainly based on clinical symptoms rather than based on protecting vulnerable groups at risk.

KEY MESSAGES

- Dutch GPs’ main motives (not) to test for pertussis were ‘confirmation of the diagnosis’ and ‘patient’s request.’
- Dutch GPs were more likely to test for pertussis in symptom-based vignettes compared to vignettes on vulnerable groups at risk.
- We recommend that GPs focus pertussis testing more on protecting vulnerable groups at risk.

## Introduction

In 2012, when preparing the current study, the highest number of pertussis cases were notified by the Netherlands (*n* = 12 868). This represents 30% of all pertussis notifications in Europe and 70% of all reported diseases in the Netherlands [1,2]. Pertussis infections can develop severely in unimmunized infants with the highest rates of complications and death [3]. Young infants are the most vulnerable group at risk as they are not yet fully protected with vaccination by the national immunization programme. Maternal vaccination and preventive measures in close contacts can protect these young infants.

However, pertussis infections are underdiagnosed and underreported as the clinical diagnosis of pertussis is difficult because of often unspecific symptoms as well as co-infection with respiratory diseases. Additionally, pertussis infections are often diagnosed too late and therefore pertussis notifications are often reported too late for public health authorities to take any preventive measures [4,5].

According to the national guidelines, pertussis testing and medical treatment is recommended when pertussis is suspected in patients living with infants aged under one-year who are not vaccinated, or not completely vaccinated, with women who are more than 34 weeks pregnant or with children who have severe heart or lung disease in the household [6].

Of all healthcare providers, general practitioners (GPs) request most (81%) of diagnostic tests for pertussis [4]. It was shown that a substantial number (16%) of GPs do not test for pertussis at all and a similar proportion does not recognize the clinical symptoms in a standardized adolescent case-patient [7]. Currently, it is largely unknown why GPs decide to test specifically for pertussis or refrain from doing so, and whether they base these decisions on clinical symptoms of patients or their proximity to vulnerable groups at risk.

Therefore, we evaluated diagnostic practices reported by GPs using an online questionnaire to improve pertussis surveillance and control. In addition, we assessed GPs’ reasons for (not) testing and intentions towards pertussis testing, whether these intentions are in line with national guidelines and possible associations with GP characteristics.

## Pertussis vaccination in the Netherlands

The Netherlands provides an extensive national immunization programme (NIP) free of charge to protect all children against 12 infectious diseases, including pertussis [3]. The introduction of general pertussis vaccination in the 1950s has tremendously reduced its incidence [8]. Currently, the efficacy of pertussis vaccines has been debated because of waning immunity, incomplete protection of infants younger than five months of age, genetic changes in *Bordetella pertussis* and the limited duration of protection [9–11]. Maternal immunization is recommended by the WHO, the ECDC and the Health Council of the Netherlands [1,12,13], and vaccines will be offered to pregnant women from the end of 2019 onwards in the Netherlands. As yet, maternal vaccination is not part of routine care or guidelines in the Netherlands. In addition, the Dutch Health Council has recommended vaccinating healthcare workers who have contact with infants younger than six months old [14].

## Methods

### Study design and setting

All GPs in the study area (*n* = 594) were invited by email to complete an online questionnaire, they were asked to reflect on their clinical diagnosis and diagnostic testing of pertussis patients in the year 2013. A reminder email was sent to non-responding GPs after three weeks. This survey study was part of a larger study aimed at evaluating pertussis surveillance and control in a distinct geographical region of the Netherlands, using reported pertussis testing practices of general practitioners, laboratory diagnostic data and public health service notifications [4,15]. This pilot survey study was conducted in Limburg, the southernmost province of the Netherlands. Its population of 1.1 million is comparable to the rest of the Netherlands in terms of sex composition and urbanization, although it is slightly older [16]. The medical ethics committee of MUMC + approved this study (14-4-060) and waived the need for informed consent.

### General practitioner questionnaire

Apart from questions on the demographics of the GP, the questionnaire was divided in three parts. The first part comprised questions on the estimated number of clinical diagnoses (based on clinical history), number of laboratory diagnoses (based on medical microbiology laboratory testing), reports of additional laboratory diagnostic testing after a clinical diagnosis and total patients tested during the previous year.

The second part was on reasons for (not) testing for pertussis. This included two multiple-choice questions. The GPs had to check one or more options from each provided lists of possible reasons and could give one other reason for testing and for not testing.

Finally, we measured the intention to test for pertussis by presenting GPs with eight clinical vignettes on a five-point scale (−2 ‘definitely not’, −1 ‘probably not’, 0 ‘possibly’, 1 ‘likely’, 2 ‘definitely’). These vignettes were developed based on the national GP guideline by infectious disease specialists and GPs [6]. For analyses, these clinical pertussis vignettes were divided into three different groups: one typical pertussis case vignette, four symptom-based vignettes and three vignettes of vulnerable groups at risk (Table 1). The clinical pertussis vignettes were ordered mixed in the questionnaire, GPs were unaware of the three groups of vignettes. The mean scores of the symptom-based vignettes and the vignettes of vulnerable groups at risk were calculated.

**Table 1.** General practitioners’ reported reasons for testing and reasons for not testing for pertussis, the Netherlands, 2013 (*n* = 122).

	% (*n*)
Reasons for testing	
To confirm the clinical pertussis diagnosis	70 (86)
On patients’ request	57 (70)
To rule out pertussis	51 (62)
To give more information on the duration of symptoms	50 (61)
To start preventive measures	43 (53)
To start treatment	7 (8)
Reasons for not testing	
The diagnostic test result does not influence treatment	70 (86)
No direct contact with risk groups (hence no need for treatment)	52 (64)
Clear diagnosis due to direct link to a confirmed pertussis case	41 (50)
Clinical assessment alone is sufficient to diagnose pertussis	33 (40)
Costs for patients	28 (34)
Treatment has already started	25 (31)
Collecting blood samples in children is too stressful	25 (31)
Child is fully vaccinated	17 (21)
Time to receive test result	16 (20)
Referral to specialist	16 (19)

### Statistical analyses

To evaluate GP-reported diagnostic practices as well as reasons for (not) testing and intentions towards pertussis testing among GPs, we analysed the data using descriptive statistics, paired *t*-tests and chi-squared tests where appropriate. Analyses were performed using SPSS package version 21.0 (IBM Inc., Somers, New York, USA).

## Results

### Study population

Of the 594 invited GPs, 122 (21%) completed the questionnaire. Respondents were on average 48 years old (SD = 9.4), were mostly male (61%), worked in a practice with on average 3336 patients (SD = 1712.1) and had 18 years of clinical experience (SD = 9.7). Forty-one percent worked full-time. In comparison, the density of GPs was 4.1 FTE per 10 000 patients in the Netherlands (with 5068 practices and 16.8 million inhabitants this equates to a mean of 3315 patients per GP-practice), Dutch GPs are on average 49 years old, 55% are male and 40% work full-time [17].

GPs reported sending their pertussis samples mainly to the six medical microbiology laboratories in the study region, while only a minority of 7% (9/122) reported sending their tests primarily to laboratories outside the region.

### GP reported pertussis diagnostic practices

On average, GPs reported to annually diagnose 6.1 (SD = 7.0, range: 0–40) pertussis cases and 84% (*n* = 103) of the GPs reported to have had at least one diagnosed case of pertussis in the previous year. In case of a clinical pertussis diagnosis (*n* = 93), 88% (*n* = 82) of GPs indicated that they requested a confirmatory laboratory test. Of all GPs, 14% (*n* = 17) did not perform any pertussis diagnostic testing during the study year, while 39% (*n* = 47) of the GPs reported to request one to four tests and 48% (*n* = 58) of the GPs reported to request five or more tests (mean = 5.9, SD = 6.5, range = 0–30). GP characteristics were similar for the groups of testing and non-testing GPs.

### Reported reasons for testing and not testing for pertussis

GPs’ reported reasons for testing and reasons for not testing for pertussis can be found in Table 1. When asked about their considerations to perform diagnostic testing for pertussis, the most frequently mentioned reasons to test were to confirm the clinical pertussis diagnosis (70%), on patients’ request (57%), to rule out pertussis (51%), to give more information on the duration of symptoms such as coughing (50%) or to start preventive measures (43%). The most important reported reasons for not testing was that GPs considered the diagnostic test result not to influence the treatment (70%) and when there is no direct contact with risk groups and hence no need for treatment (52%).

### Intentions to test for pertussis

The clinical vignette in which GPs indicated to be most likely to perform diagnostic testing for pertussis was the typical pertussis case vignette presented (a patient with two weeks of persistent coughing which had direct contact with a person diagnosed with pertussis; 64%), (Table 2). GPs reported that they were more likely to perform laboratory testing for pertussis in the symptom-based vignettes than in the vignettes of vulnerable groups at risk.

**Table 2.** General practitioners’ reported intention to test for pertussis, the Netherlands, 2013 (*n* = 122).

Intention to test^a^	Mean (SD)	% (*n*) likely or definitely
Typical pertussis case vignette: A patient with two weeks of persistent coughing who had direct contact with a person diagnosed with pertussis	0.7 (1.1)	64 (78)
Symptom-based vignettes:	0.5 (0.8)	57 (70)
• Patient with a cough and a ‘whooping’ inhalation	0.6 (1.1)	57 (70)
• Patient with frequent expiratory coughing	0.6 (1.0)	53 (65)
• Patient who vomits after coughing	0.5 (1.1)	49 (60)
• Patient with a persistent cough for more than six weeks without other symptoms	0.3 (1.0)	46 (56)
Vulnerable groups at risk vignettes:	−0.1 (0.9)	25 (30)
• Infants of three months to one year old with two weeks of persistent coughing	−0.1 (1.0)	30 (37)
• A pregnant woman (third trimester) with two weeks of persistent coughing	−0.1 (1.0)	29 (35)
• An adult with two weeks of persistent coughing who has a baby of two months old	−0.2 (1.1)	26 (32)

a Likelihood to test case ranging from −2 = definitely not, −1 = probably not, 0 = possibly, 1 = likely, 2 = definitely.

Frequent testers (GPs performing five or more laboratory pertussis tests annually) reported to be more likely to test based on vulnerable groups at risk (mean: 0.1, SD = 0.9) compared to non-frequent testers (mean: −0.3, SD = 0.8, *p* = 0.006). Male GPs reported to be more likely to test based on vulnerable groups at risk (mean: 0.0, SD = 0.9) compared to female GPs (mean: −0.3, SD = 0.7, *p* = 0.023). No differences were found for other GP characteristics (age, work experience and size of the general practice).

## Discussion

### Main findings

Of all GPs, 14% did not perform any pertussis diagnostic testing during the study year. GP characteristics were similar for the groups of testing and non-testing GPs. Our findings in terms of GPs’ reasons (not) to test indicate that they mainly test for confirmation of the diagnoses and on patient’s request. In contrast to national guidelines, GPs reported being more likely to test in the symptom-based vignettes compared to the vignettes of vulnerable groups at risk. Frequent testers and male GPs seem to test more in line with the guidelines.

### Strengths and limitations

As no medical records were available for research purposes, we collected self-reported data for this pilot study. The use of these self-reported data may introduce recall bias, whereas the numbers depicted in this study are estimates rather than exact figures. The online questionnaire of this study reported on the year of 2013. However, as pertussis testing guidelines have not changed, we consider our results are still applicable and of value for current daily practice in primary care.

In general, the use of vignettes can positively affect the construct, internal and external validity of survey studies [18]. The vignettes were clearly written and designed in cooperation with infectious diseases specialists and GPs to resemble a realistic decision-making process. Whether the reported answers accurately reflect GPs’ actual clinical practice is not entirely known from our study. However, studying this in an observational study without clinical vignettes will have ethical (concerning confidential health data), practical (costs and feasibility) and other scientific limitations (observer effects) [18].

Moreover, our study may be prone to selection bias due to the low response rate of 21%. As GPs who are more aware and evolved in pertussis testing could be more prone to participate, this could influence the generalizability of our results. The response rate was only slightly lower than other questionnaire studies in GPs [19,20] and the characteristics of responding GPs were comparable to the demographic characteristics (age, gender, percentage working fulltime and average size of general practice) of Dutch GPs in general [17].

### Comparison with existing literature

Our findings suggest that GPs mostly decide whether to test for pertussis based on clinical symptoms rather than on vulnerable groups at risk. According to the national guidelines, pertussis testing is recommended when pertussis is suspected in patients living with infants aged under 1-year-old who are not or not completely vaccinated, with women who are more than 34 weeks pregnant or with children who have severe heart or lung disease in the household [6].

While GPs report that they rarely test to start treatment, it is generally recommended to perform laboratory testing before beginning treatment. However, in a possible index case whose family includes unvaccinated or incompletely vaccinated infants <1 year of age, a woman > 34 weeks pregnant or a child with severe heart or lung disease, treatment is indicated for all family members and could start before laboratory confirmation of the index case [6,21].

Our study is unique as there is only one comparable study known on the diagnostic practices for pertussis reported in primary care. This study among a sample of American GPs, reported the diagnostic practices for pertussis in adolescents [7]. The proportion of non-testing GPs and the intention to test a typical pertussis case was similar in this study. Reasons for not testing for pertussis differed from our study as delay in obtaining test results was reported by 52% compared to 16% in our study [7].

### Implications for practice

As the GPs reported to test mainly for confirmation of the diagnosis and on patients’ request and because they reported being more likely to test for pertussis based on clinical symptoms, we recommend focus pertussis testing practices of GPs more on vulnerable groups at risk in accordance with national guidelines. GPs need to be aware that it is recommended to consider testing in families with incompletely vaccinated infants <1 year of age, women > 34 weeks pregnant and children with severe comorbidities such as heart or lung disease. Therefore, they are advised to check whether patients with symptoms suggestive of pertussis have vulnerable groups at risk in their household. As such, GPs could play a more prominent role in contact tracing (now carried out by the public health authorities in the Netherlands) to minimize the disease burden in infants.

## Conclusion

Our findings in terms of GPs’ reasons (not) to test show that they mainly test for confirmation of the diagnoses and on patient’s request. In contrast to national GP guidelines in the Netherlands, GPs reported being more likely to test in the symptom-based vignettes compared to the vignettes of vulnerable groups at risk. We recommend that GPs focus pertussis testing more on protecting vulnerable groups at risk.
